# Supplementary material for: Temporal and geographic patterns of stab injuries in young people: a retrospective cohort study from a UK major trauma centre
Source: BMJ Open. 2018 Nov 6;8(10):e023114. doi: 10.1136/bmjopen-2018-023114 (PMC6231558; doi:10.1136/bmjopen-2018-023114)
Supplement: Author's manuscript [file bmjopen-2018-023114.draft_revisions.pdf]

BMJ Open is committed to open peer review. As part of this commitment we make the peer review history of every article we publish publicly available.

When an article is published we post the peer reviewers' comments and the authors' responses online. We also post the versions of the paper that were used during peer review. These are the versions that the peer review comments apply to.

The versions of the paper that follow are the versions that were submitted during the peer review process. They are not the versions of record or the final published versions. They should not be cited or distributed as the published version of this manuscript.

BMJ Open is an open access journal and the full, final, typeset and author-corrected version of record of the manuscript is available on our site with no access controls, subscription charges or pay-per-view fees (http://bmjopen.bmj.com).

If you have any questions on BMJ Open's open peer review process please email
info.bmjopen@bmj.com

BMJ Open

Stab injuries in young people occur in characteristic temporal and geographic patterns: a retrospective cohort study from a UK major trauma centre

Journal:	*BMJ Open*
Manuscript ID	bmjopen-2018-023114
Article Type:	Research
Date Submitted by the Author:	22-Mar-2018
Complete List of Authors:	Vulliamy, Paul; Queen Mary University of London, Centre for Trauma Sciences<br>Faulkner, Mark; London Ambulance Service NHS Trust<br>Kirkwood, Graham; Newcastle University, Institute of Health and Society<br>West, Anita; Barts Health NHS Trust<br>O'Neill, Breda; Barts Health NHS Trust<br>Griffiths, Martin; Barts Health NHS Trust<br>Moore, Fionna; South East Coast Ambulance Service NHS Foundation Trust<br>Brohi, Karim; Queen Mary University of London, Centre for Trauma Sciences; Barts Health NHS Trust
Keywords:	TRAUMA MANAGEMENT, PUBLIC HEALTH, Violence Reduction, PAEDIATRICS
Note: The following files were submitted by the author for peer review, but cannot be converted to PDF. You must view these files (e.g. movies) online.	
Supplemental Video.mp4	

SCHOLARONE™
Manuscripts

TITLE PAGE

Manuscript Title: **Stab injuries in young people occur in characteristic temporal and geographic patterns: a retrospective cohort study from a UK major trauma centre**

Authors

Paul Vulliamy[1], Mark Faulkner[2], Graham Kirkwood[3], Anita West[4], Breda O'Neill[4], Martin P Griffiths[4], Fionna Moore[5], Karim Brohi[1,4]

Affiliations

[1] Centre for Trauma Sciences, Blizard Institute, Queen Mary University of London, E1 2AT

[2] London ambulance service NHS trust, 9-20 Pocock Street, London SE1 0BW

[3] Institute of Health and Society, Newcastle University, Newcastle-upon-Tyne, NE2 4AX

[4] The Royal London Hospital, Barts Health NHS Trust, Whitechapel, E1 1BB

[5] South East Coast Ambulance service NHS trust, Nexus house, Crawley, RH10 9BG

Correspondence:

Correspondence to: Dr Paul Vulliamy

Address: Centre for Trauma Sciences, Blizard Institute of Cell and Molecular Science, Whitechapel, London, England, E1 1BB

Phone: +44 208 7882 2483

Email: p.vulliamy@qmul.ac.uk

Manuscript Word Count: 2,005

For peer review only

Competing Interests Statement

All authors have completed the ICMJE uniform disclosure form at www.icmje.org/coi_disclosure.pdf and declare: no support from any organisation for the submitted work; no financial relationships with any organisations that might have an interest in the submitted work in the previous three years; no other relationships or activities that could appear to have influenced the submitted work

Copyright/license to publication

The corresponding author (P.Vulliamy) has the right to grant on behalf of all authors and does grant on behalf of all authors, an exclusive on a worldwide basis to the BMJ Publishing Group Ltd to permit this article (if accepted) to be published in BMJ editions and any other BMJPGL products and sublicences such use and exploit all subsidiary rights, as set out in our licence.

Transparency Statement

The lead author affirms that this manuscript is an honest, accurate, and transparent account of the study being reported; that no important aspects of the study have been omitted; and that any discrepancies from the study as planned have been explained.

Contributors

PV, FM and KB conceived the study. PV, MF, AW, GK, BON, MPG and KB contributed to data collection and analysis. PV and KB wrote the initial draft of the manuscript. All authors contributed to critical revisions of subsequent manuscript drafts and approve of the final version.

Funding Statement

No specific funding was received for this study.

For peer review only

ABSTRACT

Objectives: To describe the epidemiology of assaults resulting in stab injuries among young people. We hypothesised that there are specific patterns and risk factors for injury in different age groups.

Design: Eleven-year retrospective cohort study

Setting: Urban major trauma centre in the United Kingdom.

Participants: 1,824 patients under the age of 25 presenting to hospital after a stab injury resulting from assault.

Outcomes: Incident timings and locations were obtained from ambulance service records and triangulated with prospectively collected demographic and injury characteristics recorded in our hospital trauma registry. We used geospatial mapping of individual incidents to investigate the relationships between demographic characteristics and incident timing and location.

Results: The majority of stabbings occurred in males from deprived communities, with a sharp increase in incidence between the ages of 14 and 18 years. With increasing age, injuries occurred progressively later in the day (r^2=0.66, $p<0.01$) and were less frequent within 5km of home (r^2=0.59, $p<0.01$). Among children (age <16), a significant peak in injuries occurred between 4-6pm, accounting for 22% (38/172) of injuries in this group compared to 11% (182/1652) of injuries in young adults. In children, stabbings occurred earlier on school days (hours from 8am: 11.1 vs non-school day 13.7, $p<0.01$) and a greater proportion were within 5km of home (90% vs non-school day 74%, $p=0.02$). Mapping individual incidents demonstrated that the spike in frequency in the late afternoon and early evening was attributable to incidents occurring on school days and close to home.

Conclusions: Age, gender and deprivation status are potent influences on the risk of violent injury in young people. Stab injuries occur in characteristic temporal and geographical patterns according to age group, with the immediate after-school period associated with a spike in incident frequency in children. This represents an opportunity for targeted prevention strategies in this population.

For peer review only

ARTICLE SUMMARY

Strengths and limitations of this study

- Large long-term study focusing on an important public health issue in age groups at the highest risk of knife violence
- Unlike most previous studies on this topic, this study combines incident-level data on location and timing of assaults with demographic data and clinical outcomes, which allows a detailed analysis of the epidemiology of knife violence in specific age groups
- This study does not provide an insight into the patterns of stab injuries over time because of changes to the trauma system which occurred during the study period
- The generalisability of these findings to other settings and other forms of interpersonal violence may be limited

For peer review only

MANUSCRIPT TEXT

Introduction

Interpersonal violence involving knives is a major public health problem.[1] Reports of high-profile incidents are rarely absent from mainstream media, although these represent a fraction of the overall incidence. In 2017, 36,998 offences involving knives or other sharp implements were reported in England and Wales, a rise of 26% compared with the previous year.[2] Beyond the immediate physical consequences of knife violence, the psychological and social impact on individuals and communities is substantial.[3,4] Despite intensive efforts at prevention, the incidence of knife crime has increased in recent years.[2,5-6] A detailed understanding of the risk factors for stab injury in specific groups is essential to inform ongoing preventative initiatives.

Young people are the most frequent victims of knife violence.[7] Male teenagers from deprived communities in urban areas are at particularly high risk, with a peak in incidence between the ages of 16-24 years.[1-2,8] Stab injuries in children are less common than in adolescents, but also predominantly affect those living in areas with the highest level of socioeconomic deprivation.[9] The extent to which the timing, location and outcomes of stab injuries vary with age is unknown. A detailed breakdown of the epidemiology of stab injuries in different age groups would identify opportunities for targeted prevention measures in individual populations.

The objective of this study was to characterise the epidemiology of stab injuries among different age groups. We hypothesised that stabbings in children occur in specific temporal and geographic patterns which are distinct from those involving adolescents and young adults.

Methods

Study design

We performed a retrospective cohort study of patients presenting to an urban major trauma centre in London, UK. The study was approved by a regional research ethics committee (reference 15/SC/0547). All patients who met criteria for trauma team activation between 2004-2014 were screened for inclusion. We included patients under the age of 25 who presented to the emergency department after an injury involving a knife or other sharp implement. Accidental injuries and those resulting from deliberate self-harm were excluded. Demographic data,

For peer review only

injury characteristics and outcomes were recorded prospectively by a dedicated trauma nurse practitioner. Incident time and location were obtained retrospectively from the regional ambulance service database. Death preventability was determined by local peer review.

Definitions

To investigate age-group specific characteristics, we subdivided the cohort according to the World Health Organisation definitions of childhood (<16 years), late adolescence (16-19 years) and young adulthood (20-24 years).[10] Deprivation status was determined using the index of multiple deprivation score (IMD) based on home postcode and classified into quintiles according to nationally-defined cut-offs. Patients with missing data for home address were excluded from these analyses.

Data Analysis

Data were analysed using Microsoft Excel v15.3 (Microsoft, CA) and Prism v6.0 (GraphPad, CA). Maps were generated using Tableau v10.1 (Tableau software, WA). Distances between postcodes were calculated using open access online software (freemaptools.com). Continuous data are reported as median with interquartile range and were compared with Mann-Whitney U tests or Kruskal-Wallis tests with post-hoc corrections for multiple comparisons. Categorical data are displayed as number and percentage and were compared with Fisher's exact test. A two-tailed p value less than 0·05 was considered significant throughout.

Patient and Public Involvement

No patients were involved in the research design and no patients were directly involved in the study. Since the conception of this study we have established a network of patients and public representatives who help guide our ongoing violence reduction initiatives and will be involved in dissemination of these findings to communities and other institutions.

Results

Between 2004-2014, 3,274 victims of assault resulting in penetrating trauma presented to the emergency department at our institution, of whom 1,824 (56%) were aged under 25 and were included in the analysis. Of these, 172 (9.4%) were children, 861 (47.2%) were aged 16-19 and 791 (43.4%) were aged 20-24 **(table 1)**. The

For peer review only

locations of individual incidents over time is shown in **supplemental video 1.** A substantial majority of patients (1127/1594, 71%) were from the most deprived quintile while only 1% (15/1594) were from the least deprived quintile, excluding 230 patients who had no recorded home postcode. No major demographic differences were identified across the three age groups, but there was a trend towards higher in-hospital mortality in the paediatric group (7/172, 4.1%) compared to the adolescent and young adult group (26/1652, 1.9%, p=0·08) despite comparable injury severity scores. Preventable or potentially preventable deaths at peer review were significantly more frequent in paediatric patients compared to older adolescents and young adults (5/7 deaths in the paediatric group vs 6/38 in those aged ≥16, p=0.001).

Table 1: Demographics of the study cohort. Values are median with interquartile range unless stated. P-values compare age groups with Kruskal-Wallis test for continuous data and chi-squared test for categorical data.

	All Patients	<16 years	16-19 years	20-24 years	
**Number of Patients**	**1,824**	**172**	**861**	**791**	**p-value**
Patient characteristics					
Age, years	19 (17-21)	15 (14-15)	17 (17-18)	22 (20-23)	<0.001
Male, n (%)	1772 (97%)	169 (98%)	847 (98%)	756 (96%)	0.002
Index of Multiple Deprivation[1]	42 (32-50)	44 (33-50)	43 (33-50)	41 (31-50)	0.38
Most deprived quintile, n (%)[1]	1127/1594 (71%)	116/157 (74%)	564/776 (73%)	447/661 (68%)	0.06
Injury Characteristics					
Injury Severity Score	2 (1-9)	1 (1-9)	2 (1-9)	2 (1-9)	0.39
Severe injury[2]	225 (12%)	24 (14%)	99 (12%)	102 (13%)	0.51
Multiple injuries	947 (52%)	81 (47%)	456 (53%)	410 (52%)	0.37
Hospital admission	1116 (61%)	103 (62%)	548 (64%)	465 (59%)	0.13
Length of stay, days[3]	2 (1-4)	2 (1-4)	2 (1-4)	2 (1-4)	0.61
In-hospital mortality	39 (2%)	7 (4%)	12 (1%)	20 (2%)	0.05

[1]Excluding 230 patients with missing home postcodes
[2]Injury Severity Score >15
[3]Hospital admissions only

Distinct patterns of injury were observed across the age range within the study cohort. The frequency of stab injuries rose sharply in the late teenage years, reaching a peak at age 18 before gradually declining (**figure 1A**). The severity of physical injury did not vary with age and the majority of injuries were classified as non-critical (**table 1** and **figure 1B**). There were significant differences in the timing and location of injuries, with younger patients tending to be stabbed earlier in the day and closer to home (**figure 1C and 1D**).

For peer review only

To investigate these variations further, we compared patterns of injury in children (<16) with older adolescents and young adults (16-24). Among children, a significant peak in frequency occurred between 16:00-18:00, accounting for 22% of all injuries in this age group as compared to 11% in young adults (p<0.01**, figure 2A**). Young adults were significantly more likely to be stabbed after midnight (16-24 years old 31% vs <16 years old 16%, p<0.01). We next examined the distance from home address to incident in these two age groups and found distinct distributions (**figure 2B**). A large proportion of incidents occurred within 1km of home in both children (35%) and young adults (41%, p=0.08). Children were significantly more likely to be stabbed between 1-5km from home (48% vs 35%, p=0.002) but less likely to be stabbed more than 5km from home (16% vs 25%, p=0·04).

We hypothesized that the specific pattern of injuries in children was related to school attendance. We therefore subdivided incidents into those occurring on school days and those occurring during school holidays or at weekends. Children were more likely to be stabbed on a school day than the older age group (58% vs 50%, p=0.06). In the paediatric group, stabbings occurred earlier on a school day (hours from 8am: school day 11.1 [8.6 to 13.9] vs non-school day 13.7 [9.7 to 15.9], p<0.01; **figure 2C)** and a greater proportion were within 5km of home **(**school day 90% vs non-school day 74%, p=0.02**; figure 2D).** Mapping individual incidents demonstrated that in children the spike in frequency in the late afternoon and early evening was attributable to incidents occurring on school days **(figure 3).** The majority of stabbings in this time frame on school days occurred within 5km of home, which encompasses the average distance from home to school in children living in London.[11] On non-school days, incidents in children were similar to those in young adults in terms of the timing (hours from 8am: 13.7 [9.7 to 15.9] vs 13.9 [10.1-17.5], p=0.22) and location relative to home (proportion >5km from home 25.8% vs 26.4%, p=1.0). Stabbings on school days within 5km of home accounted for 47% of the total injury burden among children, compared to 33% in young adults (p<0.001).

Discussion

In this long-term retrospective cohort study, we have shown that assaults resulting in penetrating injuries occur in distinct age-related patterns. Specifically, the period immediately after school accounts for a large proportion

For peer review only - http://bmjopen.bmj.com/site/about/guidelines.xhtml

of incidents in children, and these predominantly occur close to home and school. This represents an opportunity for targeted preventative strategies in this population.

At both an individual and community level, knife crime has major physical, psychological and social consequences.[1,12] The overall incidence of interpersonal violence involving knives is increasing in the United Kingdom and anecdotal reports suggest that assaults resulting in multiple injuries from multiple assailants are also on the rise.[2,7] However, multimodal preventative strategies can produce dramatic reductions in weapons offenses and injuries among young people.[13] This is exemplified by the success of violence reduction initiatives in Glasgow, which have resulted in consistent and substantial decreases in knife crime in a country labelled the most violent in the developed world by the United Nations in 2005.[14,15] Aggressive law enforcement formed the initial basis of this programme, including legislation to impose mandatory sentences for knife possession, increased duration of sentences and widespread use of the 'stop and search' strategy. In isolation, such strategies have had limited effect in other settings and may increase tensions between communities and law enforcement.[16,17] Crucially, in the Glasgow example this approach is coupled to a range of educational and behavioural programmes which raise awareness of the consequences of knife violence.[13] Addressing the broader socioeconomic factors associated with violence is another cornerstone of many successful prevention initiatives. Neighbourhood deprivation and concentrated poverty have consistently been shown to be independent risk factors for involvement in violent crime,[18] an association which is clearly reflected in our cohort. There is ample evidence that community-based interventions to reduce environmental contributors to violence and minimise inequality can reduce the incidence of offending, violent injury, and incarceration among young people.[19-21]

Our results provide detailed age-specific information regarding the timing and location of stab injuries, which have a number of potential implications for targeted violence reduction strategies. The sharp increase in stab injuries between the ages of 14 to 16 suggests that educational programmes and other preventative interventions are best delivered in primary or early secondary education. Given the peak in incidents at the end of the school day, an attractive option is staggered release times from school which could be coupled to a visible deterrent by law enforcement at transport hubs, eateries and other areas of pupil congregation after school. Combating weapons carriage through a 'stop and search' strategy, which remains a hotly debated issue, will be better informed by accurate incident data such as that presented here. However, these direct approaches will only produce a sustained reduction in knife crime when delivered in the context of a co-ordinated plan to combat violent behaviour and its root causes.

For peer review only

From a clinical perspective, the majority of stab injuries in our cohort resulted in relatively minor physical injuries and deaths were infrequent. However, over half of all stabbings resulted in multiple injuries. This is more than double the frequency observed in a study conducted in our catchment area thirty years ago,[22] which supports anecdotal observations of increasing intensity of violence involving knives. We found that children had a higher overall mortality and a higher frequency of potentially preventable deaths compared to young adults despite comparable injury severity scores. Further study is required to determine the reasons for this observation and whether a similar trend is evident in other trauma systems and in other injury patterns. Although the implementation of regional trauma networks in England and Wales has produced substantial improvements in outcomes from major injury in adults, similar evidence in children is currently lacking and is the subject of an ongoing multicentre investigation.[23]

The limitations to our study include its observational design, most notably in that the apparent relationship between paediatric stab injuries and school attendance represents an association only. The generalisability of our findings to other geographical areas and other forms of interpersonal violence may be limited. Owing to the healthcare-based setting of this study we were not able to consider the assailants as well as the victims in this analysis. Finally, because of changes in our regional trauma system during the study period, we were not able to comment on trends in incidence over time.

Our study illustrates and reiterates the potent influence of deprivation, age and gender on the risk of violent injury. We have demonstrated that there are age-specific epidemiological patterns of stabbings among young people and identify specific targets for focused violence reduction strategies in children. Delivery of co-ordinated violence reduction interventions are essential to drive sustained reductions in interpersonal violence and will be better informed by the recognition of knife crime as a pressing public health issue.

References

1. World Health Organisation. European report on preventing violence and knife crime among young people. 2017. http://www.euro.who.int/__data/assets/pdf_file/0012/121314/E94277.pdf

2. Office for National Statistics. Crime in England and Wales: year ending June 2017. 2017. https://www.ons.gov.uk/peoplepopulationandcommunity/crimeandjustice/bulletins/crimeinenglandandwales/june2017

3. Flannry DJ, Singer MI, Wester K. Violence exposure, psychological trauma, and suicide risk in a community sample of dangerously violent adolescents. *J Am Acad Child Adolesc Psychiatry*. 2001;40(4):435-42.

4. Weaver CM, Borkowski JG, Whitman TL. Violence Breeds Violence: Childhood Exposure and Adolescent Conduct Problems. *J Community Psychol*. 2008;36(1):96-112.

5. HM Government. Violent Crime Reduction Act (2006). https://www.legislation.gov.uk/ukpga/2006/38

6. Crewdson K, Lockey D, Weaver A, Davies GE. Is the prevalence of deliberate penetrating trauma increasing in London? Experiences of an urban pre-hospital trauma service. *Injury* 2009;40:560–563.

7. Pallett JR, Sutherland E, Glucksman E, Tunnicliff M, Keep JW. A cross-sectional study of knife injuries at a London major trauma centre. *Ann R Coll Surg Engl*. 2014;96(1):23-6.

8. Apps JR, Williams C, McGuinness A, Gabbie S, Sutcliffe AG. Stabbing and safeguarding in children and young people: a Pan-London service evaluation and audit. *JRSM Short Rep*. 2013;4(7):1-7.

9. Melling L, Lansdale N, Mullassery D, Taylor-Robinson D, Jesudason EC. Penetrating assaults in children: often non-fatal near-miss events with opportunities for prevention in the UK. *Injury*. 2012;43(12):2088-93

10. World Health Organisation. Youth and health risks: Report from the 64th World Health Assembly. 2011. http://apps.who.int/gb/ebwha/pdf_files/WHA64/A64_25-en.pdf

11. HM Government, Department of Transport. National Travel Survey 2014. https://www.gov.uk/government/uploads/system/uploads/attachment_data/file/476635/travel-to-school.pdf

12. Krug EG, Mercy JA, Dahlberg LL, Zwi AB. The world report on violence and health. *Lancet*. 2002;360(9339):1083-8.

13. Kath Murray, Paul McGuinness, Michele Burman, Susan McVie, Evaluation of the Whole System Approach to Young People who Offend in Scotland. Scottish Government, 2015.

14. Dijk, J. van, J. van Kesteren, P. Smit. Criminal Victimisation in International Perspective; Key findings from the 2004-2005 ICVS and EU ICS. 2007. http://www.unicri.it/services/library_documentation/publications/icvs/publications/ICVS2004_05report.pdf

15. Rainey SR, Simpson J, Page S, et al. The impact of violence reduction initiatives on emergency department attendance. *Scott Med J*. 2015;60(2): 90–94

16. Braga AA. Hot spots policing and crime prevention: a systematic review of randomized controlled trials. *J Exp Criminol* 2005;1:317-42.

17. Chris Eades, Roger Grimshaw, Arianna Silvestri and Enver Solomon. 'Knife crime'. A review of evidence and policy (2nd Edition). *Centre for Crime and Justice Studies* (2007)

18. Lesley McAra , Susan McVie. Understanding youth violence: The mediating effects of gender, poverty and vulnerability. *Journal of Criminal Justice* 2016;45:71–77

19. Casteel, C., Peek-Asa, C., 2000. Effectiveness of crime prevention through environ- mental design (CPTED) in reducing robberies. *Am J Prev Med*. 2000;18(4,Suppl 1):99–115.

20. Cerda, M., et al., 2012. Reducing violence by transforming neighborhoods: a natural experiment in Medellin, Colombia. *Am J Epidemiol*. 2012;175(10):1045–1053.

21. Cassidy T, Inglis G, Wiysonge C, Matzopoulos R. A systematic review of the effects of poverty deconcentration and urban upgrading on youth violence. *Health Place*. 2014;26:78-87.

For peer review only

22. Stebbings WS, Chalstrey LJ, Gilmore OJ, Shand WS, Staunton MD, Thomson JP. Stab injury--the experience of an East London Hospital 1978-1983. *Postgrad Med J*. 1987;63(736):81-4.
23. Cole E, Lecky F, West A, Smith N, Brohi K, Davenport R; ELoTS Study Collaborators. The Impact of a Pan-regional Inclusive Trauma System on Quality of Care. *Ann Surg*. 2016;264(1):188-94.

Figure Legends

Figure 1: Age-related variations in the pattern of stab injury. A: Number of cases. B: Injury severity score. Box plots depict median, interquartile range and 10-90th percentiles. C: Time of injury. D: Proportion of incidents occurring within 5km of home. Linear regression line and 95% confidence intervals shown in solid and dashed lines respectively.

Figure 2: Injuries in children occur in characteristic patterns and are related to school attendance. A: Timing of injuries in children (red) and young adults (blue). B: Distance from home address to incident in children and young adults. C: Timing of injuries on school days as compared to weekends or school holidays. D: Location of injuries on school days as compared to weekends or school holidays. *p<0.05, **p<0.01, ***p<0.001, Kruskal-Wallis test with Dunn's multiple comparisons test.

Figure 3: Incident locations, timing and distance from home in children on school days and non-school days. Circle size is inversely proportional to the distance from incident to home address. Colours indicate incident timing.

Supplemental Video: Stab injuries 2004-2014. Each frame represents incidents occurring per quarter year. Dots represent individual incident locations. Fatal injuries are represented by black dots.

For peer review only

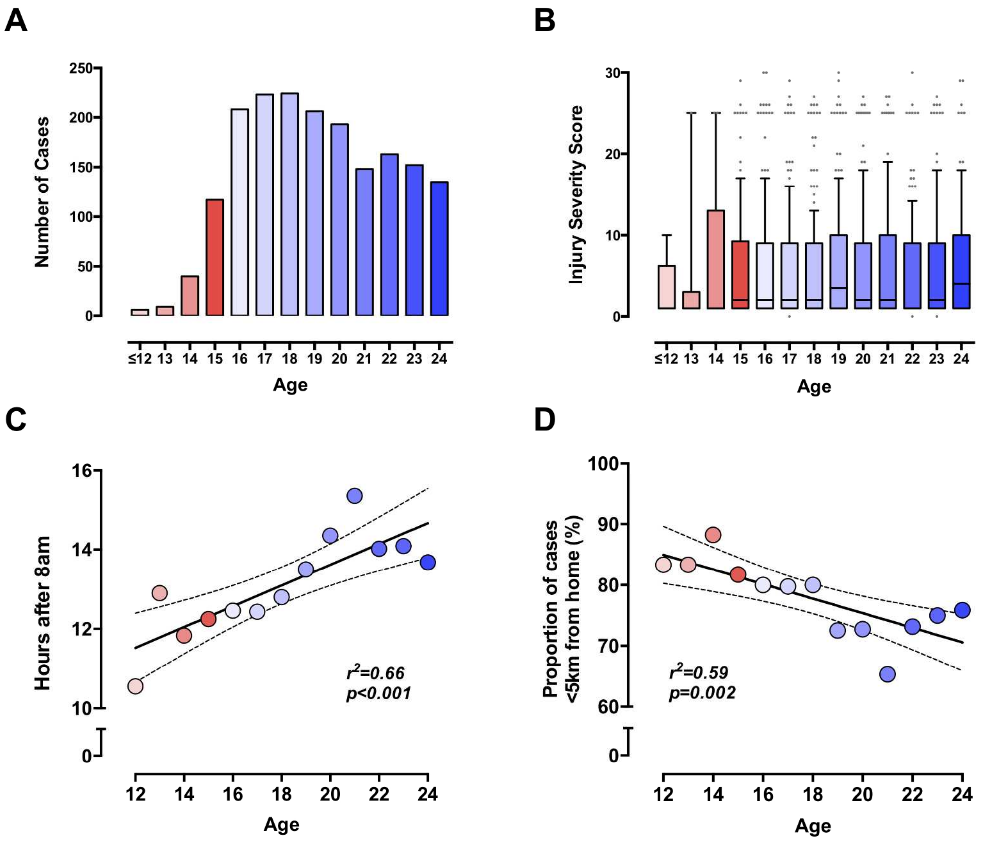

Figure 1: Age-related variations in the pattern of stab injury. A: Number of cases. B: Injury severity score. Box plots depict median, interquartile range and 10-90th percentiles. C: Time of injury. D: Proportion of incidents occurring within 5km of home. Linear regression line and 95% confidence intervals shown in solid and dashed lines respectively.

218x188mm (300 x 300 DPI)

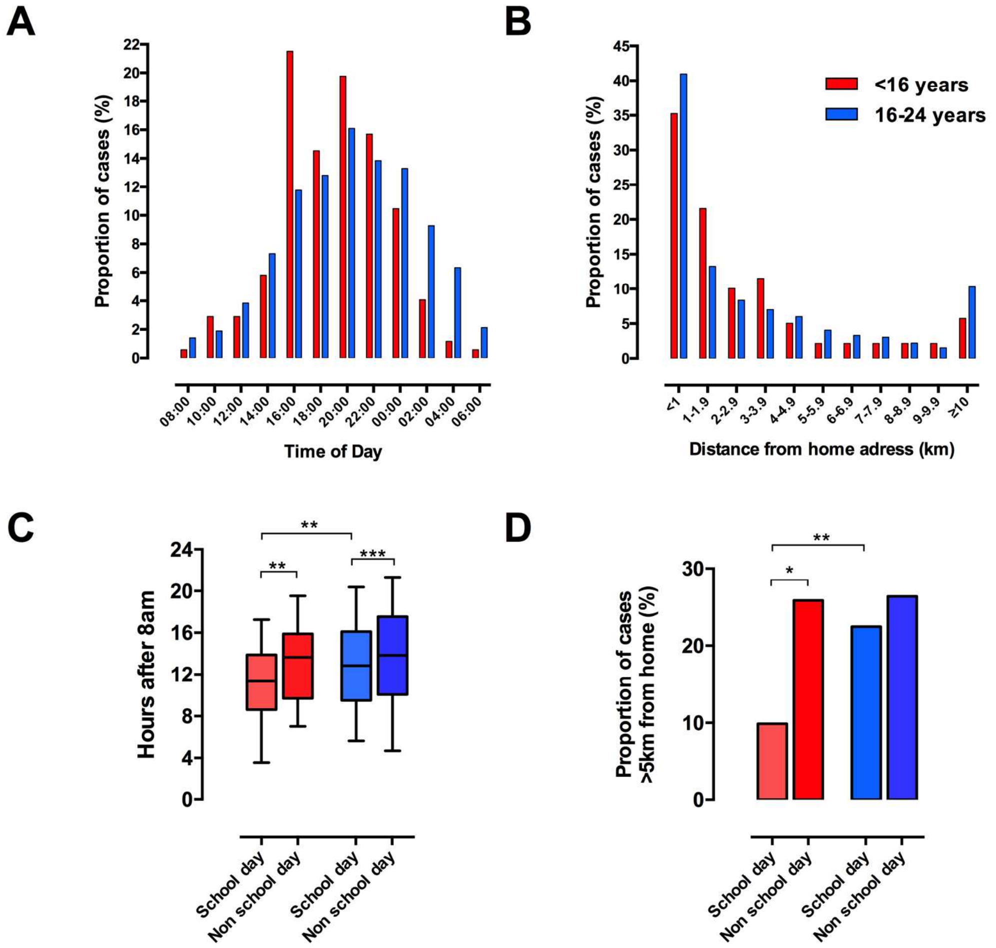

Figure 2: Injuries in children occur in characteristic patterns and are related to school attendance. A: Timing of injuries in children (red) and young adults (blue). B: Distance from home address to incident in children and young adults. C: Timing of injuries on school days as compared to weekends or school holidays. D: Location of injuries on school days as compared to weekends or school holidays. *p<0.05, **p<0.01, ***p<0.001, Kruskal-Wallis test with Dunn's multiple comparisons test.

175x172mm (300 x 300 DPI)

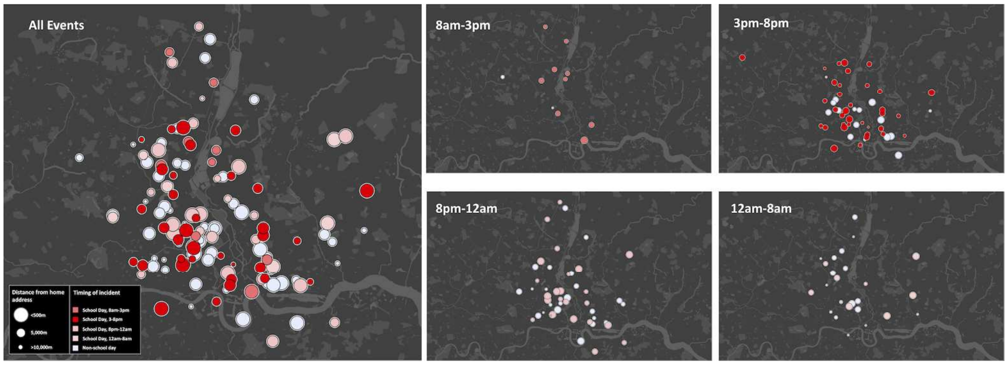

Figure 3: Incident locations, timing and distance from home in children on school days and non-school days. Circle size is inversely proportional to the distance from incident to home address. Colours indicate incident timing.

175x62mm (300 x 300 DPI)

For peer review only

STROBE Statement—Checklist of items that should be included in reports of ***cohort studies***

Study Title: **Stab injuries in young people occur in characteristic temporal and geographic patterns: a retrospective cohort study from a UK major trauma centre**

	Item No	Recommendation
**Title and abstract**	1	(*a*) Indicate the study's design with a commonly used term in the title or the abstract **(Title)**
		(*b*) Provide in the abstract an informative and balanced summary of what was done and what was found **(Abstract)**
**Introduction**		
Background/rationale	2	Explain the scientific background and rationale for the investigation being reported **(Introduction)**
Objectives	3	State specific objectives, including any prespecified hypotheses **(intro para3)**
**Methods**		
Study design	4	Present key elements of study design early in the paper (**Title; Methods, para 1**)
Setting	5	Describe the setting, locations, and relevant dates, including periods of recruitment, exposure, follow-up, and data collection (**Title; Methods, para 1)**
Participants	6	(*a*) Give the eligibility criteria, and the sources and methods of selection of participants. Describe methods of follow-up (**Methods, para 1)**
		(*b*) For matched studies, give matching criteria and number of exposed and unexposed **N/A**
Variables	7	Clearly define all outcomes, exposures, predictors, potential confounders, and effect modifiers. Give diagnostic criteria, if applicable (**Methods, para 1)**
Data sources/ measurement	8*	For each variable of interest, give sources of data and details of methods of assessment (measurement). Describe comparability of assessment methods if there is more than one group **Methods para 1**
Bias	9	Describe any efforts to address potential sources of bias **(Methods, para 1)**
Study size	10	Explain how the study size was arrived at **Not done**
Quantitative variables	11	Explain how quantitative variables were handled in the analyses. If applicable, describe which groupings were chosen and why (**Methods para 2 and 3)**
Statistical methods	12	(*a*) Describe all statistical methods, including those used to control for confounding **(Methods para 3)**
		(*b*) Describe any methods used to examine subgroups and interactions (**Methods para 2 and 3)**
		(*c*) Explain how missing data were addressed **(Methods para 2)**
		(*d*) If applicable, explain how loss to follow-up was addressed **N/A**
		(*e*) Describe any sensitivity analyses **N/A**
**Results**		
Participants	13*	(a) Report numbers of individuals at each stage of study—eg numbers potentially eligible, examined for eligibility, confirmed eligible, included in the study, completing follow-up, and analysed (**Results, para 1)**
		(b) Give reasons for non-participation at each stage **N/A**
		(c) Consider use of a flow diagram **Not done**
Descriptive data	14*	(a) Give characteristics of study participants (eg demographic, clinical, social) and information on exposures and potential confounders (**Results, Para 1; Table 1**)

For peer review only

		(b) Indicate number of participants with missing data for each variable of interest (**Results, para 1; table 1)**
		(c) Summarise follow-up time (eg, average and total amount) **N/A**
Outcome data	15*	Report numbers of outcome events or summary measures over time (**Results, para 1)**
Main results	16	(*a*) Give unadjusted estimates and, if applicable, confounder-adjusted estimates and their precision (eg, 95% confidence interval). Make clear which confounders were adjusted for and why they were included **Results**
		(*b*) Report category boundaries when continuous variables were categorized (**Methods, para 2; Table 1**)
		(*c*) If relevant, consider translating estimates of relative risk into absolute risk for a meaningful time period **N/A**
Other analyses	17	Report other analyses done—eg analyses of subgroups and interactions, and sensitivity analyses (**Results, Para 3 and 4)**
**Discussion**		
Key results	18	Summarise key results with reference to study objectives **(Discussion, para 1)**
Limitations	19	Discuss limitations of the study, taking into account sources of potential bias or imprecision. Discuss both direction and magnitude of any potential bias **Discussion, para 5)**
Interpretation	20	Give a cautious overall interpretation of results considering objectives, limitations, multiplicity of analyses, results from similar studies, and other relevant evidence **Discussion, para 2-5)**
Generalisability	21	Discuss the generalisability (external validity) of the study results **Discussion, para 5)**
**Other information**		
Funding	22	Give the source of funding and the role of the funders for the present study and, if applicable, for the original study on which the present article is based **N/A**

For peer review only

For peer review only - http://bmjopen.bmj.com/site/about/guidelines.xhtml

BMJ Open

Temporal and geographic patterns of stab injuries in young people: a retrospective cohort study from a UK major trauma centre

Journal:	*BMJ Open*
Manuscript ID	bmjopen-2018-023114.R1
Article Type:	Research
Date Submitted by the Author:	09-Aug-2018
Complete List of Authors:	Vulliamy, Paul; Queen Mary University of London, Centre for Trauma Sciences<br>Faulkner, Mark; London Ambulance Service NHS Trust<br>Kirkwood, Graham; Newcastle University, Institute of Health and Society<br>West, Anita; Barts Health NHS Trust<br>O'Neill, Breda; Barts Health NHS Trust<br>Griffiths, Martin; Barts Health NHS Trust<br>Moore, Fionna; South East Coast Ambulance Service NHS Foundation Trust<br>Brohi, Karim; Queen Mary University of London, Centre for Trauma Sciences; Barts Health NHS Trust
**Primary Subject Heading**:	Public health
Secondary Subject Heading:	Epidemiology, Paediatrics
Keywords:	TRAUMA MANAGEMENT, PUBLIC HEALTH, Violence Reduction, PAEDIATRICS
Note: The following files were submitted by the author for peer review, but cannot be converted to PDF. You must view these files (e.g. movies) online.	
Supplemental Video.mp4	

SCHOLARONE™
Manuscripts

TITLE PAGE

Manuscript Title: Temporal and geographic patterns of stab injuries in young people: a retrospective cohort study from a UK major trauma centre

Authors

Paul Vulliamy[1], Mark Faulkner[2], Graham Kirkwood[3], Anita West[4], Breda O'Neill[4], Martin P Griffiths[4], Fionna Moore[5], Karim Brohi[1,4]

Affiliations

[1] Centre for Trauma Sciences, Blizard Institute, Queen Mary University of London, E1 2AT

[2] London ambulance service NHS trust, 9-20 Pocock Street, London SE1 0BW

[3] Institute of Health and Society, Newcastle University, Newcastle-upon-Tyne, NE2 4AX

[4] The Royal London Hospital, Barts Health NHS Trust, Whitechapel, E1 1BB

[5] South East Coast Ambulance service NHS trust, Nexus house, Crawley, RH10 9BG

Correspondence:

Correspondence to: Dr Paul Vulliamy

Address: Centre for Trauma Sciences, Blizard Institute of Cell and Molecular Science, Whitechapel, London, England, E1 1BB

Phone: +44 208 7882 2483

Email: p.vulliamy@qmul.ac.uk

Manuscript Word Count: 2,161

For peer review only

Competing Interests Statement

All authors have completed the ICMJE uniform disclosure form at www.icmje.org/coi_disclosure.pdf and declare: no support from any organisation for the submitted work; no financial relationships with any organisations that might have an interest in the submitted work in the previous three years; no other relationships or activities that could appear to have influenced the submitted work

Copyright/license to publication

The corresponding author (P.Vulliamy) has the right to grant on behalf of all authors and does grant on behalf of all authors, an exclusive on a worldwide basis to the BMJ Publishing Group Ltd to permit this article (if accepted) to be published in BMJ editions and any other BMJPGL products and sublicences such use and exploit all subsidiary rights, as set out in our licence.

Transparency Statement

The lead author affirms that this manuscript is an honest, accurate, and transparent account of the study being reported; that no important aspects of the study have been omitted; and that any discrepancies from the study as planned have been explained.

Contributors

PV, FM and KB conceived the study. PV, MF, AW, GK, BON, MPG and KB contributed to data collection and analysis. PV and KB wrote the initial draft of the manuscript. All authors contributed to critical revisions of subsequent manuscript drafts and approve of the final version.

Funding Statement

No specific funding was received for this study.

Data sharing statement

No additional data available.

For peer review only

ABSTRACT

Objectives: To describe the epidemiology of assaults resulting in stab injuries among young people. We hypothesised that there are specific patterns and risk factors for injury in different age groups.

Design: Eleven-year retrospective cohort study

Setting: Urban major trauma centre in the United Kingdom.

Participants: 1,824 patients under the age of 25 presenting to hospital after a stab injury resulting from assault.

Outcomes: Incident timings and locations were obtained from ambulance service records and triangulated with prospectively collected demographic and injury characteristics recorded in our hospital trauma registry. We used geospatial mapping of individual incidents to investigate the relationships between demographic characteristics and incident timing and location.

Results: The majority of stabbings occurred in males from deprived communities, with a sharp increase in incidence between the ages of 14 and 18 years. With increasing age, injuries occurred progressively later in the day (r^2=0.66, p<0.01) and were less frequent within 5km of home (r^2=0.59, p<0.01). Among children (age <16), a significant peak in injuries occurred between 4-6pm, accounting for 22% (38/172) of injuries in this group compared to 11% (182/1652) of injuries in young adults. In children, stabbings occurred earlier on school days (hours from 8am: 11.1 vs non-school day 13.7, p<0.01) and a greater proportion were within 5km of home (90% vs non-school day 74%, p=0.02). Mapping individual incidents demonstrated that the spike in frequency in the late afternoon and early evening was attributable to incidents occurring on school days and close to home.

Conclusions: Age, gender and deprivation status are potent influences on the risk of violent injury in young people. Stab injuries occur in characteristic temporal and geographical patterns according to age group, with the immediate after-school period associated with a spike in incident frequency in children. This represents an opportunity for targeted prevention strategies in this population.

For peer review only

ARTICLE SUMMARY

Strengths and limitations of this study

- Large long-term study focusing on an important public health issue in age groups at the highest risk of knife violence
- Unlike most previous studies on this topic, this study combines incident-level data on location and timing of assaults with demographic data and clinical outcomes, which allows a detailed analysis of the epidemiology of knife violence in specific age groups
- This study does not provide an insight into the patterns of stab injuries over time because of changes to the trauma system which occurred during the study period
- The generalisability of these findings to other settings and other forms of interpersonal violence may be limited

For peer review only

MANUSCRIPT TEXT

Introduction

Interpersonal violence involving knives is a major public health problem.[1] Reports of high-profile incidents are rarely absent from mainstream media, although these represent a fraction of the overall incidence. In 2017, 36,998 offences involving knives or other sharp implements were reported in England and Wales, a rise of 26% compared with the previous year.[2] Beyond the immediate physical consequences of knife violence, the psychological and social impact on individuals and communities is substantial.[3,4] Despite intensive efforts at prevention, the incidence of knife crime has increased in recent years.[2,5-6] A detailed understanding of the risk factors for stab injury in specific groups is essential to inform ongoing preventative initiatives.

Young people are the most frequent victims of knife violence.[7] Male teenagers from deprived communities in urban areas are at particularly high risk, with a peak in incidence between the ages of 16-24 years.[1-2,8] Stab injuries in children are less common than in adolescents, but also predominantly affect those living in areas with the highest level of socioeconomic deprivation.[9] The extent to which the timing, location and outcomes of stab injuries vary with age is unknown. A detailed breakdown of the epidemiology of stab injuries in different age groups would identify opportunities for targeted prevention measures in individual populations.

The objective of this study was to characterise the epidemiology of stab injuries among different age groups. We hypothesised that stabbings in children occur in specific temporal and geographic patterns which are distinct from those involving adolescents and young adults.

Methods

Study design and setting

We performed a retrospective cohort study of patients presenting to an urban major trauma centre in London, UK. Our hospital receives approximately 3000 patients requiring trauma team activations per year, of which 25% are now penetrating injuries. The trauma service covers a population of around 3.5 million people and encompasses some of the most deprived regions in the country. All patients who met criteria for trauma team activation between 2004-2014 were screened for inclusion. We included patients under the age of 25 who

For peer review only

presented to the emergency department after an injury involving a knife or other sharp implement. Accidental injuries and those resulting from deliberate self-harm were excluded. Demographic data, injury characteristics and outcomes were recorded prospectively by a dedicated trauma nurse practitioner. Incident time and location were obtained retrospectively from the regional ambulance service database. Death preventability was determined by local peer review. The study was approved by a regional research ethics committee (reference 15/SC/0547).

Definitions

To investigate age-group specific characteristics, we subdivided the cohort according to the World Health Organisation definitions of childhood (<16 years), late adolescence (16-19 years) and young adulthood (20-24 years).[10] Deprivation status was determined using the index of multiple deprivation score (IMD) based on home postcode and classified into quintiles according to nationally-defined cut-offs. Patients with missing data for home address were excluded from these analyses.

Data Analysis

Data were analysed using Microsoft Excel v15.3 (Microsoft, CA) and Prism v6.0 (GraphPad, CA). Maps were generated using Tableau v10.1 (Tableau software, WA). Distances between postcodes were calculated using open access online software (freemaptools.com). Continuous data are reported as median with interquartile range and were compared with Mann-Whitney U tests or Kruskal-Wallis tests with post-hoc corrections for multiple comparisons. Categorical data are displayed as number and percentage and were compared with Fisher's exact test. A two-tailed p value less than 0.05 was considered significant throughout.

Patient and Public Involvement

No patients were involved in the research design and no patients were directly involved in the study. Since the conception of this study we have established a network of patients and public representatives who help guide our ongoing violence reduction initiatives and will be involved in dissemination of these findings to communities and other institutions.

For peer review only

Results

Between 2004-2014, 3,274 victims of assault resulting in penetrating trauma presented to the emergency department at our institution, of whom 1,824 (56%) were aged under 25 and were included in the analysis. Of these, 172 (9.4%) were children, 861 (47.2%) were aged 16-19 and 791 (43.4%) were aged 20-24 (table 1). The locations of individual incidents over time is shown in supplemental video 1. The annual number of presentations increased by an average of 25% each year. A substantial majority of patients (1127/1594, 71%) were from the most deprived quintile while only 1% (15/1594) were from the least deprived quintile, excluding 230 patients who had no recorded home postcode. No major demographic differences were identified across the three age groups, but there was a trend towards higher in-hospital mortality in the paediatric group (7/172, 4.1%) compared to the adolescent and young adult group (26/1652, 1.9%, p=0.08) despite comparable injury severity scores. Preventable or potentially preventable deaths at peer review were significantly more frequent in paediatric patients compared to older adolescents and young adults (5/7 deaths in the paediatric group vs 6/38 in those aged ≥16, p=0.001).

For peer review only

Table 1: Demographics of the study cohort. Values are median with interquartile range unless stated. P-values compare age groups with Kruskal-Wallis test for continuous data and chi-squared test for categorical data.

	All Patients	<16 years	16-19 years	20-24 years	
**Number of Patients**	**1,824**	**172**	**861**	**791**	**p-value**
Patient characteristics					
Age, years	19 (17-21)	15 (14-15)	17 (17-18)	22 (20-23)	<0.001
Male, n (%)	1772 (97%)	169 (98%)	847 (98%)	756 (96%)	0.002
Index of Multiple Deprivation[1]	42 (32-50)	44 (33-50)	43 (33-50)	41 (31-50)	0.38
Most deprived quintile, n (%)[1]	1127/1594 (71%)	116/157 (74%)	564/776 (73%)	447/661 (68%)	0.06
Injury Characteristics					
Injury Severity Score	2 (1-9)	1 (1-9)	2 (1-9)	2 (1-9)	0.39
Severe injury[2]	225 (12%)	24 (14%)	99 (12%)	102 (13%)	0.51
Multiple injuries	947 (52%)	81 (47%)	456 (53%)	410 (52%)	0.37
Hospital admission	1116 (61%)	103 (62%)	548 (64%)	465 (59%)	0.13
Length of stay, days[3]	2 (1-4)	2 (1-4)	2 (1-4)	2 (1-4)	0.61
In-hospital mortality	39 (2%)	7 (4%)	12 (1%)	20 (2%)	0.05

[1]Excluding 230 patients with missing home postcodes
[2]Injury Severity Score >15
[3]Hospital admissions only

Distinct patterns of injury were observed across the age range within the study cohort. The frequency of stab injuries rose sharply in the late teenage years, reaching a peak at age 18 before gradually declining (figure 1A). The severity of physical injury did not vary with age and the majority of injuries were classified as non-critical (table 1**)**. There were significant differences in the timing and location of injuries, with younger patients tending to be stabbed earlier in the day and closer to home (figure 1B and 1C).

To investigate these variations further, we compared patterns of injury in children (<16) with older adolescents and young adults (16-24). Among children, a significant peak in frequency occurred between 16:00-18:00, accounting for 22% of all injuries in this age group as compared to 11% in young adults ($p<0.01$**,** figure 2A). Young adults were significantly more likely to be stabbed after midnight (16-24 years old 31% vs <16 years old 16%, $p<0.01$). We next examined the distance from home address to incident in these two age groups and found distinct distributions (figure 2B). A large proportion of incidents occurred within 1km of home in both children

For peer review only

(35%) and young adults (41%, p=0.08). Children were significantly more likely to be stabbed between 1-5km from home (48% vs 35%, p=0.002) but less likely to be stabbed more than 5km from home (16% vs 25%, p=0.04).

We hypothesized that the specific pattern of injuries in children was related to school attendance. We therefore subdivided incidents into those occurring on school days and those occurring during school holidays or at weekends. Children were more likely to be stabbed on a school day than the older age group (58% vs 50%, p=0.06). In the paediatric group, stabbings occurred earlier on a school day (hours from 8am: school day 11.1 [8.6 to 13.9] vs non-school day 13.7 [9.7 to 15.9], p<0.01; figure 2C) and a greater proportion were within 5km of home (school day 90% vs non-school day 74%, p=0.02; figure 2D). Mapping individual incidents demonstrated that in children the spike in frequency in the late afternoon and early evening was attributable to incidents occurring on school days (figure 3). The majority of stabbings in this time frame on school days occurred within 5km of home, which encompasses the average distance from home to school in children living in London.[11] On non-school days, incidents in children were similar to those in young adults in terms of the timing (hours from 8am: 13.7 [9.7 to 15.9] vs 13.9 [10.1-17.5], p=0.22) and location relative to home (proportion >5km from home 25.8% vs 26.4%, p=1.0). Stabbings on school days within 5km of home accounted for 47% of the total injury burden among children, compared to 33% in young adults (p<0.001).

Discussion

In this long-term retrospective cohort study, we have shown that assaults resulting in penetrating injuries occur in distinct age-related patterns. Specifically, the period immediately after school accounts for a large proportion of incidents in children, and these predominantly occur close to home and school. This represents an opportunity for targeted preventative strategies in this population.

At both an individual and community level, knife crime has major physical, psychological and social consequences.[1,12] The incidence of interpersonal violence involving knives has progressively increased in the United Kingdom in recent years, reaching a seven-year high in 2017, and anecdotal reports suggest that assaults resulting in multiple injuries from multiple assailants are also on the rise.[2,7] However, multimodal preventative strategies can produce dramatic reductions in weapons offenses and injuries among young people.[13] This is

exemplified by the success of violence reduction initiatives in Glasgow, which have resulted in consistent and substantial decreases in knife crime in a country labelled the most violent in the developed world by the United Nations in 2005.[14,15] Aggressive law enforcement formed the initial basis of this programme, including legislation to impose mandatory sentences for knife possession, increased duration of sentences and widespread use of the 'stop and search' strategy. In isolation, such strategies have had limited effect in other settings and may increase tensions between communities and law enforcement.[16,17] Crucially, in the Glasgow example this approach is coupled to a range of educational and behavioural programmes which raise awareness of the consequences of knife violence.[13] Addressing the broader socioeconomic factors associated with violence is another cornerstone of many successful prevention initiatives. Neighbourhood deprivation and concentrated poverty have consistently been shown to be independent risk factors for involvement in violent crime,[18] an association which is clearly reflected in our cohort. There is ample evidence that community-based interventions to reduce environmental contributors to violence and minimise inequality can reduce the incidence of offending, violent injury, and incarceration among young people.[19-21]It is clear that a multifaceted approach with sustained investment from government and the community is required for effective violence reduction.[22,23]

Our results provide detailed age-specific information regarding the timing and location of stab injuries, which have a number of potential implications for targeted violence reduction strategies. The sharp increase in stab injuries between the ages of 14 to 16 suggests that educational programmes and other preventative interventions are best delivered in primary or early secondary education. Given the peak in incidents at the end of the school day, an attractive option is staggered release times from school which could be coupled to a visible deterrent by law enforcement at transport hubs, eateries and other areas of pupil congregation after school. Combating weapons carriage through a 'stop and search' strategy, which remains a hotly debated issue, will be better informed by accurate incident data such as that presented here. However, these direct approaches will only produce a sustained reduction in knife crime when delivered in the context of a co-ordinated plan to combat violent behaviour and its root causes.

From a clinical perspective, the majority of stab injuries in our cohort resulted in relatively minor physical injuries and deaths were infrequent. However, over half of all stabbings resulted in multiple injuries. This is more than double the frequency observed in a study conducted in our catchment area thirty years ago,[24] which supports anecdotal observations of increasing intensity of violence involving knives. We found that children had a higher overall mortality and a higher frequency of potentially preventable deaths compared to young adults

For peer review only - http://bmjopen.bmj.com/site/about/guidelines.xhtml

despite comparable injury severity scores. Further study is required to determine the reasons for this observation and whether a similar trend is evident in other trauma systems and in other injury patterns. Although the implementation of regional trauma networks in England and Wales has produced substantial improvements in outcomes from major injury in adults, similar evidence in children is currently lacking and is the subject of an ongoing multicentre investigation.[25]

The limitations to our study include its observational design, most notably in that the apparent relationship between paediatric stab injuries and school attendance represents an association only. The generalisability of our findings to other geographical areas and other forms of interpersonal violence may be limited. Because we only included patients meeting criteria for trauma team activation, our study may have missed those attending with more minor injuries. Owing to the healthcare-based setting of this study we were not able to consider the demographics of the assailants as well as the victims in this analysis. In addition, our registry did not allow detailed analysis of behavioural patterns, violent recidivism and gang involvement in individual patients. These gaps in the present study are currently being investigated prospectively within our trauma system. Finally, because of changes in our regional trauma system during the study period, we were not able to comment on trends in incidence over time.

Our study illustrates and reiterates the potent influence of deprivation, age and gender on the risk of violent injury. We have demonstrated that there are age-specific epidemiological patterns of stabbings among young people and identify specific targets for focused violence reduction strategies in children. Long-term, multi-agency interventions are essential to drive sustained reductions in interpersonal violence and will be better informed by the recognition of knife crime as a pressing public health issue.

For peer review only

References

1. World Health Organisation. European report on preventing violence and knife crime among young people (2017). http://www.euro.who.int/__data/assets/pdf_file/0012/121314/E94277.pdf. Accessed June 2018.

2. Office for National Statistics. Crime in England and Wales: year ending June 2017. https://www.ons.gov.uk/peoplepopulationandcommunity/crimeandjustice/bulletins/crimeinenglandandwales/june2017. Accessed June 2018.

3. Flannry DJ, Singer MI, Wester K. Violence exposure, psychological trauma, and suicide risk in a community sample of dangerously violent adolescents. *J Am Acad Child Adolesc Psychiatry*. 2001;40(4):435-42.

4. Weaver CM, Borkowski JG, Whitman TL. Violence Breeds Violence: Childhood Exposure and Adolescent Conduct Problems. *J Community Psychol*. 2008;36(1):96-112.

5. HM Government. Violent Crime Reduction Act (2006). https://www.legislation.gov.uk/ukpga/2006/38

6. Crewdson K, Lockey D, Weaver A, et al. Is the prevalence of deliberate penetrating trauma increasing in London? Experiences of an urban pre-hospital trauma service. *Injury* 2009;40:560–563.

7. Pallett JR, Sutherland E, Glucksman E, et al. A cross-sectional study of knife injuries at a London major trauma centre. *Ann R Coll Surg Engl*. 2014;96(1):23-6.

8. Apps JR, Williams C, McGuinness A, et al. Stabbing and safeguarding in children and young people: a Pan-London service evaluation and audit. *JRSM Short Rep*. 2013;4(7):1-7.

9. Melling L, Lansdale N, Mullassery D, et al. Penetrating assaults in children: often non-fatal near-miss events with opportunities for prevention in the UK. *Injury*. 2012;43(12):2088-93

10. World Health Organisation. Youth and health risks: Report from the 64th World Health Assembly. 2011. http://apps.who.int/gb/ebwha/pdf_files/WHA64/A64_25-en.pdf. Accessed June 2018.

11. HM Government, Department of Transport. National Travel Survey 2014. https://www.gov.uk/government/uploads/system/uploads/attachment_data/file/476635/travel-to-school.pdf. Accessed June 2018.

12. Krug EG, Mercy JA, Dahlberg LL, et al. The world report on violence and health. *Lancet*. 2002; 360(9339):1083-8.

13. Kath Murray, Paul McGuinness, Michele Burman, et al. Evaluation of the Whole System Approach to Young People who Offend in Scotland. Scottish Government (2015).

For peer review only

14. Dijk, J. van, J. van Kesteren, P. Smit. Criminal Victimisation in International Perspective; Key findings from the 2004-2005 ICVS and EU ICS (2007). http://www.unicri.it/services/library_documentation/publications/icvs/publications/ICVS2004_05report.pdf.

15. Rainey SR, Simpson J, Page S, et al. The impact of violence reduction initiatives on emergency department attendance. *Scott Med J*. 2015;60(2): 90–94

16. Braga AA. Hot spots policing and crime prevention: a systematic review of randomized controlled trials. *J Exp Criminol* 2005;1:317-42.

17. Chris Eades, Roger Grimshaw, Arianna Silvestri, et al. 'Knife crime'. A review of evidence and policy (2nd Edition). Centre for Crime and Justice Studies (2007).

18. Lesley McAra, Susan McVie. Understanding youth violence: The mediating effects of gender, poverty and vulnerability. *Journal of Criminal Justice* 2016; 45:71–77

19. Casteel C, Peek-Asa C, 2000. Effectiveness of crime prevention through environmental design (CPTED) in reducing robberies. *Am J Prev Med*. 2000;18(4 Suppl):99–115.

20. Cerda M, Morenoff JD, Hansen BB, et al. Reducing violence by transforming neighborhoods: a natural experiment in Medellin, Colombia. *Am J Epidemiol*. 2012;175(10):1045–1053.

21. Cassidy T, Inglis G, Wiysonge C, et al. A systematic review of the effects of poverty deconcentration and urban upgrading on youth violence. *Health Place*. 2014;26:78-87.

22. Miao TA, Umemoto K, Gonda D, et al. Essential elements for community engagement in evidence-based youth violence prevention. *Am J Community Psychol*. 2011;48(1-2):120-32

23. Vivolo AM1, Matjasko JL, Massetti GM. Mobilizing communities and building capacity for youth violence prevention: the National Academic Centers of Excellence for Youth Violence Prevention. *Am J Community Psychol*. 2011;48(1-2):141-5.

24. Stebbings WS, Chalstrey LJ, Gilmore OJ, et al. Stab injury--the experience of an East London Hospital 1978-1983. *Postgrad Med J*. 1987;63(736):81-4.

25. Cole E, Lecky F, West A, et al. ELoTS Study Collaborators. The Impact of a Pan-regional Inclusive Trauma System on Quality of Care. *Ann Surg*. 2016;264(1):188-94.

For peer review only

Figure Legends

Figure 1: Age-related variations in the pattern of stab injury. A: Number of patients by age. B: Time of injury. C: Proportion of incidents occurring within 5km of home. Linear regression line and 95% confidence intervals shown in solid and dashed lines respectively.

Figure 2: Injuries in children occur in characteristic patterns and are related to school attendance. A: Timing of injuries in children (red) and young adults (blue). B: Distance from home address to incident in children and young adults. C: Timing of injuries on school days as compared to weekends or school holidays. D: Location of injuries on school days as compared to weekends or school holidays. *p<0.05, **p<0.01, ***p<0.001, Kruskal-Wallis test with Dunn's multiple comparisons test.

Figure 3: Incident locations, timing and distance from home in children on school days and non-school days. Circle size is inversely proportional to the distance from incident to home address. Colours indicate incident timing.

Supplemental Video: Stab injuries 2004-2014. Each frame represents incidents occurring per quarter year. Dots represent individual incident locations. Fatal injuries are represented by black dots.

For peer review only

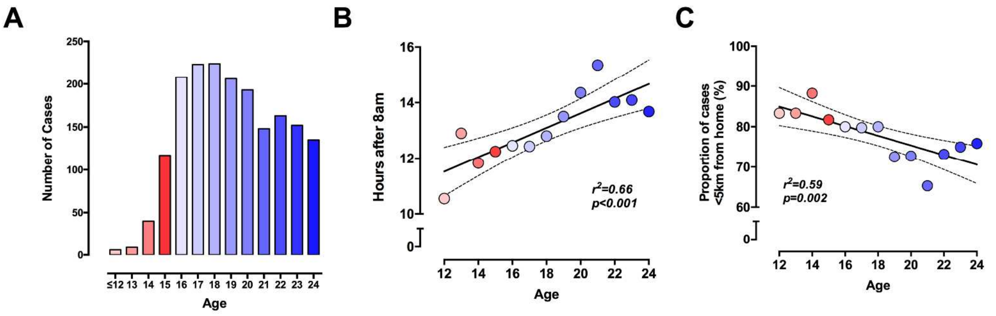

Figure 1: Age-related variations in the pattern of stab injury. A: Number of patients by age. B: Time of injury. C: Proportion of incidents occurring within 5km of home. Linear regression line and 95% confidence intervals shown in solid and dashed lines respectively.

252x83mm (300 x 300 DPI)

For peer review only

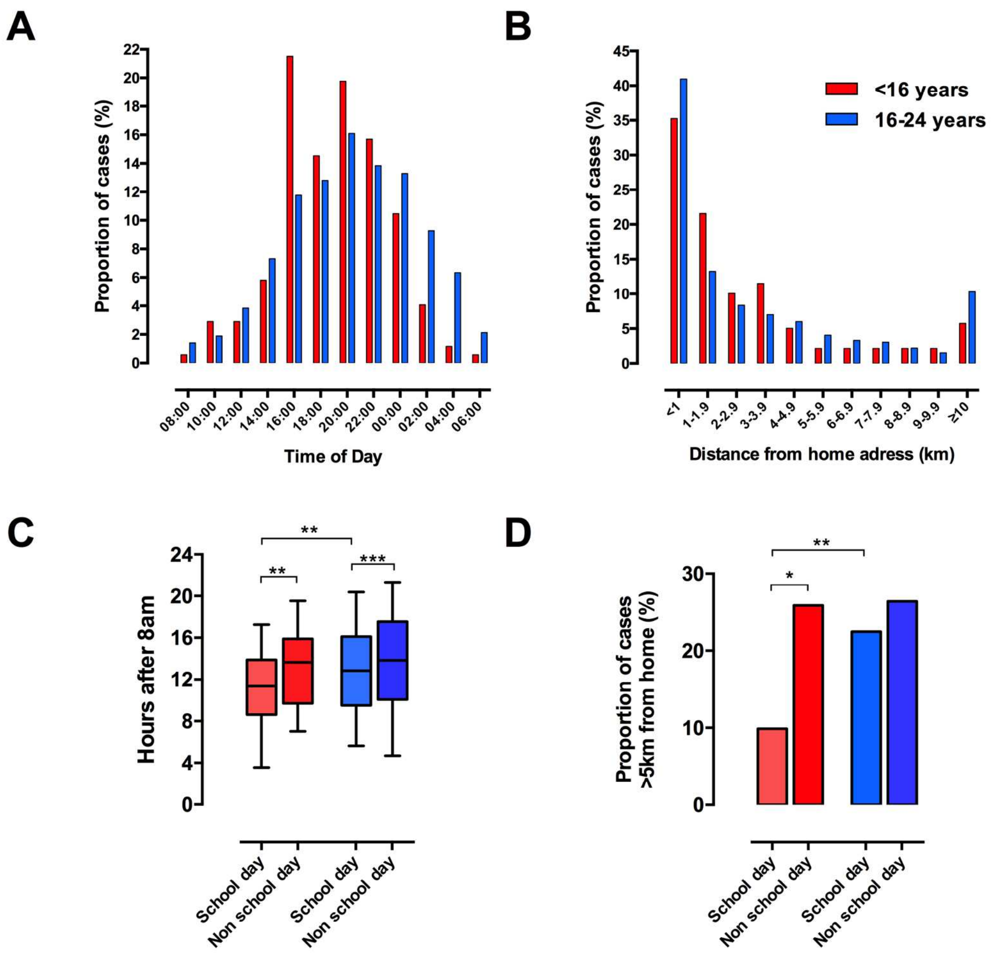

Figure 2: Injuries in children occur in characteristic patterns and are related to school attendance. A: Timing of injuries in children (red) and young adults (blue). B: Distance from home address to incident in children and young adults. C: Timing of injuries on school days as compared to weekends or school holidays. D: Location of injuries on school days as compared to weekends or school holidays. *p<0.05, **p<0.01, ***p<0.001, Kruskal-Wallis test with Dunn's multiple comparisons test.

175x172mm (300 x 300 DPI)

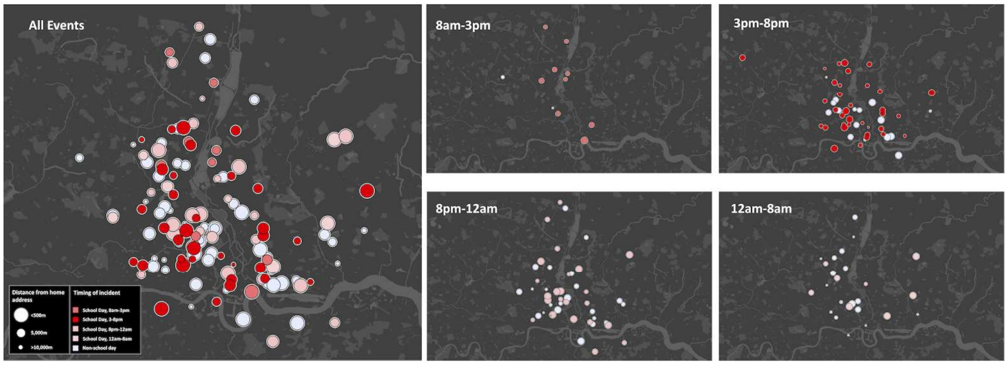

Figure 3: Incident locations, timing and distance from home in children on school days and non-school days. Circle size is inversely proportional to the distance from incident to home address. Colours indicate incident timing.

350x125mm (300 x 300 DPI)

STROBE Statement—Checklist of items that should be included in reports of ***cohort studies***

Study Title: **Stab injuries in young people occur in characteristic temporal and geographic patterns: a retrospective cohort study from a UK major trauma centre**

	Item No	Recommendation
**Title and abstract**	1	(*a*) Indicate the study's design with a commonly used term in the title or the abstract **(Title)**
		(*b*) Provide in the abstract an informative and balanced summary of what was done and what was found **(Abstract)**
**Introduction**		
Background/rationale	2	Explain the scientific background and rationale for the investigation being reported **(Introduction)**
Objectives	3	State specific objectives, including any prespecified hypotheses **(intro para3)**
**Methods**		
Study design	4	Present key elements of study design early in the paper (**Title; Methods, para 1**)
Setting	5	Describe the setting, locations, and relevant dates, including periods of recruitment, exposure, follow-up, and data collection (**Title; Methods, para 1)**
Participants	6	(*a*) Give the eligibility criteria, and the sources and methods of selection of participants. Describe methods of follow-up (**Methods, para 1)**
		(*b*) For matched studies, give matching criteria and number of exposed and unexposed **N/A**
Variables	7	Clearly define all outcomes, exposures, predictors, potential confounders, and effect modifiers. Give diagnostic criteria, if applicable (**Methods, para 1)**
Data sources/ measurement	8*	For each variable of interest, give sources of data and details of methods of assessment (measurement). Describe comparability of assessment methods if there is more than one group **Methods para 1**
Bias	9	Describe any efforts to address potential sources of bias **(Methods, para 1)**
Study size	10	Explain how the study size was arrived at **Not done**
Quantitative variables	11	Explain how quantitative variables were handled in the analyses. If applicable, describe which groupings were chosen and why (**Methods para 2 and 3)**
Statistical methods	12	(*a*) Describe all statistical methods, including those used to control for confounding **(Methods para 3)**
		(*b*) Describe any methods used to examine subgroups and interactions (**Methods para 2 and 3)**
		(*c*) Explain how missing data were addressed **(Methods para 2)**
		(*d*) If applicable, explain how loss to follow-up was addressed **N/A**
		(*e*) Describe any sensitivity analyses **N/A**
**Results**		
Participants	13*	(a) Report numbers of individuals at each stage of study—eg numbers potentially eligible, examined for eligibility, confirmed eligible, included in the study, completing follow-up, and analysed (**Results, para 1)**
		(b) Give reasons for non-participation at each stage **N/A**
		(c) Consider use of a flow diagram **Not done**
Descriptive data	14*	(a) Give characteristics of study participants (eg demographic, clinical, social) and information on exposures and potential confounders (**Results, Para 1; Table 1**)

For peer review only - http://bmjopen.bmj.com/site/about/guidelines.xhtml

		(b) Indicate number of participants with missing data for each variable of interest (**Results, para 1; table 1)**
		(c) Summarise follow-up time (eg, average and total amount) **N/A**
Outcome data	15*	Report numbers of outcome events or summary measures over time (**Results, para 1)**
Main results	16	(*a*) Give unadjusted estimates and, if applicable, confounder-adjusted estimates and their precision (eg, 95% confidence interval). Make clear which confounders were adjusted for and why they were included **Results**
		(*b*) Report category boundaries when continuous variables were categorized (**Methods, para 2; Table 1**)
		(*c*) If relevant, consider translating estimates of relative risk into absolute risk for a meaningful time period **N/A**
Other analyses	17	Report other analyses done—eg analyses of subgroups and interactions, and sensitivity analyses (**Results, Para 3 and 4)**
**Discussion**		
Key results	18	Summarise key results with reference to study objectives **(Discussion, para 1)**
Limitations	19	Discuss limitations of the study, taking into account sources of potential bias or imprecision. Discuss both direction and magnitude of any potential bias **Discussion, para 5)**
Interpretation	20	Give a cautious overall interpretation of results considering objectives, limitations, multiplicity of analyses, results from similar studies, and other relevant evidence **Discussion, para 2-5)**
Generalisability	21	Discuss the generalisability (external validity) of the study results **Discussion, para 5)**
**Other information**		
Funding	22	Give the source of funding and the role of the funders for the present study and, if applicable, for the original study on which the present article is based **N/A**

For peer review only
